# Endothelial Dysfunction and Extra-Articular Neurological Manifestations in Rheumatoid Arthritis

**DOI:** 10.3390/biom11010081

**Published:** 2021-01-10

**Authors:** Jessica Maiuolo, Carolina Muscoli, Micaela Gliozzi, Vincenzo Musolino, Cristina Carresi, Sara Paone, Sara Ilari, Rocco Mollace, Ernesto Palma, Vincenzo Mollace

**Affiliations:** 1 IRC-FSH Department of Health Sciences, University “Magna Græcia” of Catanzaro, Campus Universitario di Germaneto, 88100 Catanzaro, Italy; jessicamaiuolo@virgilio.it (J.M.); muscoli@unicz.it (C.M.); micaela.gliozzi@gmail.com (M.G.); xabaras3@hotmail.com (V.M.); carresi@unicz.it (C.C.); sara.paone06@gmail.com (S.P.); rocco.mollace@gmail.com (R.M.); palma@unicz.it (E.P.); 2 Nutramed S.c.a.r.l, Complesso Ninì Barbieri, Roccelletta di Borgia, 88021 Catanzaro, Italy; sara.ilari@hotmail.it; 3 IRCCS San Raffaele, Via di Valcannuta 247, 00133 Rome, Italy

**Keywords:** rheumatoid arthritis, inflammation, neurological extra-articular manifestations, endothelial dysfunction, polyphenols and Mediterranean diet

## Abstract

Rheumatoid arthritis (RA) is a chronic, systemic, inflammatory autoimmune disease that affects about 1% of the global population, with a female–male ratio of 3:1. RA preferably affects the joints, with consequent joint swelling and deformities followed by ankylosis. However, evidence has accumulated showing that patients suffering from RA can also develop extra-articular manifestations, including cardiovascular disease states, neuropathies, and multiorgan dysfunction. In particular, peripheral nerve disorders showed a consistent impact in the course of the disease (prevalence about 20%) mostly associated to vasculitis of the nerve vessels leading to vascular ischemia, axonal degeneration, and neuronal demyelination. The pathophysiological basis of this RA-associated microvascular disease, which leads to impairment of assonal functionality, is still to be better clarified. However, endothelial dysfunction and alterations of the so-called brain-nerve barrier (BNB) seem to play a fundamental role. This review aims to assess the potential mechanisms underlying the impairment of endothelial cell functionality in the development of RA and to identify the role of dysfunctional endothelium as a causative mechanism of extra-articular manifestation of RA. On the other hand, the potential impact of lifestyle and nutritional interventions targeting the maintenance of endothelial cell integrity in patients with RA will be discussed as a potential option when approaching therapeutic solutions in the course of the disease.

## 1. Introduction

Rheumatoid arthritis (RA) is a chronic systemic autoimmune disease, which affects approximately 1% of the global population and occurs preferably in females, with a female-to-male ratio of 3:1 [1]. The disease can begin slowly and gradually with mainly non-specific symptoms such as fatigue, low-grade fever, general malaise, and joint pain; later, more defined symptoms such as intense pain, morning stiffness, and swelling of the joints have been described [2]. In some cases, RA begins in an acute form, and in this case joint involvement with distinctive signs of inflammation occurs [3]. In general, the joints most frequently affected are those of the hands and wrists, followed by those of the feet, knees, elbows, ankles, up to involving the wider joints of the shoulders, hips, jaws, and cervical spine [4]. With overt disease, joint swelling occurs, caused by synovial effusion being associated to joint deformities and ankylosis [5]. For this reason, RA is associated with a lower quality of life, a partial and/or total incapacity to work, a form of progressive disability, and a higher probability of developing other co-morbidities [6]. The onset of the disease occurs between the ages of 25 and 50, although in recent years there has been a progressive increase in the incidence rate as the average age of onset rises from 50 to 55 years. In this case, RA affects both genders with the same incidence [7]. Recently, evidence has been accumulated that RA patients can also develop extra-articular manifestations (EAMs), which mainly affect the skin, eyes, the nervous system, the respiratory system, the gastrointestinal system, the cardiovascular system, and the kidney [8]. In particular, neurological manifestations of RA such as peripheral nerve disturbances, seem to occur very early in the course of the disease, being expressed by limb paresthesias and by motor nerve impairment which characterize the late stages of the disease, contributing to the severe disability of RA patients. [1]. The pathophysiological basis of early impairment of nerve endings in RA patients is still unknown. However, studies carried out by magnetic resonance imaging (MRI) showed the presence of abnormalities in the brain circulation of patients with RA and revealed the occurrence of vascular-associated inflammatory disorders in the brain tissue with the brain [9,10]. Thus, early vascular impairment in RA seems to play a crucial role in extra-articular manifestations of the disease, including neurological RA-associated disorders. 

The endothelium is an essential endocrine organ that participates in maintaining the homeostasis of the whole body, consisting of specialized epithelial cells that line the vascular system, lymphatic vessels, and the heart. The functions performed by the endothelium include control of the contractile state of the vascular smooth muscles and cardiomyocytes, coagulant and rheological properties of blood, adhesion, and vascular wall permeability. In addition, under physiological conditions, the endothelium is responsible for regulating the communication between blood and tissues by means of a wide spectrum of signaling molecules. The dysfunction of the endothelium, therefore, involves the loss of these homeostatic mechanisms and the onset of pathologies [11]. In particular, evidence exists that endothelial cells contribute to maintaining the nerve endings into a functional active state via the substrate and energy supply. On the other hand, endothelial cell functionality contributes to the integrity of the so-called brain-nerve barrier (BNB), which regulates the exchange of substrates and catabolites. Moreover, alterations of endothelial cells at the level of nerve microcirculation have been shown to be involved in the development of peripheral nerve diseases [12]. Thus, it is likely that neurological disfunctions seen in the course of the development of RA could play a fundamental role in extra-articular manifestations of RA. 

The aims of the present review are:

To assess the extra-articular manifestations of RA with a special focus on neurological dysfunctions accompanying the disease; 

To better clarify the role of endothelial dysfunction in the pathophysiological mechanisms of RA-related neurological dysfunction; and

To identify the potential contribution of lifestyle and nutritional changes in the therapeutic approach to RA-associated neurological disorders.

## 2. Extra-Articular Manifestations in Rheumatoid Arthritis

### 2.1. Multiorgan RA-Associated Disease

Patients with RA can develop a wide range of extra-articular manifestations (EAMs) and it seems they are caused by the involvement of genetic and immune factors [13]. In most cases, EAMs are accompanied by a process of vasculitis and a high titer of circulating Rheumatoid Factor (RF) and Anti-Citrullinated Protein Antibodies (ACPA) [14]. It has been shown that there is an increased risk of developing cardiovascular diseases in patients with RA and this percentage is about 50% higher than in healthy individuals [15]. The systemic inflammatory state present in RA can result in atherosclerotic damage, reduction of high-density lipoproteins, increased development of metabolic syndrome, and a state of hypertension [16,17,18]. In some instances, heart involvement may be asymptomatic and constitute a silent complication of RA [19].

Skin manifestations, which may occur in RA, include a wide range of diseases that have a significant negative impact on patients, on physical, emotional, and psychosocial well-being. For this reason, early recognition, understanding of these manifestations through an interdisciplinary approach, and adequate pharmacological treatment would be necessary in order to better manage this disabling condition. The major skin manifestations present in patients with RA may be rheumatoid nodules, granulomatous disorders, and neutrophilic dermatoses [20,21]. Rheumatoid nodules occur in 25–35% of patients with RA and have been associated with cardiovascular disorders [22]. In addition, the skin of these patients may also present periungual infarctions, petechiae, purpura, or erythema. Again, in this case, skin manifestations are frequently preceded by necrotizing vasculitis involving medium-sized vessels [23].

Pulmonary involvement is a common EAM of RA and occurs in about 40% of patients [24]. These diseases can affect any of the lung compartments and can manifest as a complication of RA therapy, as an opportunistic infection, or as a result of a form of drug toxicity [25]. The most frequent manifestation is interstitial pneumonia, accompanied by documented widespread alveolar damage. In particular, the identification and management of these diseases requires the combination of histopathological and radiological clinical examinations [26]. 

Gastrointestinal manifestations, which occur in conjunction with RA, are rare but can have a very negative impact on patients. Some gastrointestinal processes are directly related to RA, while others may be consequences of pharmacological treatment of concurrent autoimmune diseases. In general, these manifestations are found in most patients with a particularly severe form of RA [27]. It concerns inflammatory processes against small vessels responsible for ischemic ulcers and perforations. The main symptoms include abdominal pain, nausea, diarrhea, and malabsorption [28]. Liver involvement is more rarely observed and is characterized by alteration of liver enzyme levels [29].

Patients with RA are normally accompanied by progressive damage to the joint bone and cartilage which is related to disability over time. The impact of inflammation on bone in RA is, in fact, particularly destructive and increases the risk of developing osteopotosis [30]. Patients with RA present a marked demineralization and erosion of bones. Inflammatory events are responsible for osteoclastogenesis and reduced osteoblastogenesis: inflammatory cytokines (tumor necrosis factor-α (TNF-α), interleukin-1 (IL-1), IL-6, IL-17) and immune cells negatively impact osteoblast differentiation and their ability to produce mineralized matrix [31]. For this reason and to improve the quality of life, it would be desirable for these patients to regularly monitor bone mineral density and undergo calcium and vitamin D supplementation [32]. In recent years, enormous progress has been made in the prevention of bone loss in the RA thanks to the advent of disease-changing agents, including biological agents and small molecules, which limit inflammation and can have a direct impact on the prevention of osteoclastogenesis. However, repair of existing bone erosion, although feasible, is rarely observed [33]. As new agents are introduced to control inflammation in RA and new mechanisms are identified to affect synovitis, it may be possible in the future to completely repair the damaged bone.

### 2.2. Neurological Disorders Associated to RA

EAMs that affect patients with RA are a wide range of neuronal damage. In particular, it has been highlighted that about 20% of patients can develop neuropathies, multiple mononeuritis, distal sensory neuropathies, and sensory-motor neuropathies. In all these disorders, neurological involvement occurs as a consequence of vasculitis of the nerve vessels leading to vascular ischemia, axonal degeneration, and neuronal demyelination [34]. Many neuropathies develop due to nerve compression, as in the case of carpal tunnel syndrome, generating not only pain but also paresthesia and neuronal damage. It has been shown that chronic synovitis, at the foot level, is associated with the development of Morton neuroma and tarsal tunnel syndrome, two pathologies responsible for pain in patients with RA [35].

In addition to carpal tunnel syndrome, central nervous system involvement in RA patients includes multiple manifestations such as meningitis, optical atrophy, cerebral vasculitis, and rheumatoid nodule formation [36,37]. Among these alterations, cervical myelopathy is the most common in patients with RA for more than 15 years and is associated with significant morbidity and mortality [38]. The frequency with which cervical myelopathy occurs is 2.5% and the main symptoms are neck pain, occipital headache, sensory deficits, lower cranial nerve palsy, and transient ischemic attacks. These symptoms are caused by compression of the spinal cord and brain stem [39]. The cervical vertebrae C1 and C2 are the typical targets of the pathology and the involvement of the cervical spine can present various forms including erosions of the vertebral endplates, erosions of the spinous process, and changes in the apophyseal joint followed by osteoporosis [40]. These inflammatory lesions of the cervical spine, associated with frequent subluxations, occur within the first ten years after the diagnosis of RA, although many patients remain completely asymptomatic [41]. Diagnosis of cervical myelopathy is carried out by X-rays, through which it is possible to assess the parameters of the cranio-cervical junction. MRI also provides more detailed information on ligament structures [42]. Rheumatoid meningitis is a neurological manifestation of RA, affecting the central nervous system, which can occur during a remission phase of the autoimmune disease [43]. The main symptoms include headache, seizures, deafness, speech disturbances, and stroke-like symptoms, e.g., hemiparesis and cognitive impairment. Since these symptoms can be misinterpreted, it is necessary to make a correct diagnosis through the combination of numerous data including objective clinical presentation, analysis of the cerebrospinal fluid obtained by lumbar puncture, MRI of the brain, and a biopsy that can exclude other etiologies. Furthermore, to rule out possible infections, the cerebrospinal fluid should be negative [44]. The analysis of the pathological manifestations describes the chronic inflammation of the meninges, the concomitant presence of vasculitis, and necrotizing granulomas [45]. In some cases, but not all, the presence of ACPA and RF autoantibodies can be detected [46]. Rheumatoid nodules are nodular lesions found in the subcutaneous area normally subjected to pressure or mechanical stress, such as the joints of the fingers or forearm. They are usually present in about 20–40% of patients with more aggressive RA and the SPSA form; RF is, normally, present in patients with autoimmune disease who develop rheumatoid nodules. If, on the other hand, RF is absent it may be that the patients develop the other pathological forms [47]. Rheumatoid nodules are characterized by specific histological peculiarities: there are numerous macrophages and multinucleated cells arranged around a central necrotic area [48]. The presence of rheumatoid nodules reduces the functional capacity of the patient who is affected and a rehabilitation program is recommended. Sometimes, pharmacological and rehabilitative treatment is replaced by surgery, but this option has not always proven to be decisive [49]. In patients with RA, drug-induced toxicity is also involved in neuronal damage [50]: the scientific literature has highlighted that many extra-articular neurological manifestations present in RA could be due to adverse reactions of the common drugs taken for this autoimmune disease. Among these, it seems that prolonged use or high doses of glucocorticoids (GCs) can cause manifestations psychiatric and cognitive impairment [51]. The use of Methotrexate, a drug widely used in RA, can cause peripheral neuropathies, retinal damage, and ear alterations [52]. Extra-articular neurological manifestations in RA also include the ophthalmological field; dry eye represents the most frequent occurrence of RA in the early stages of the disease and affects one in four patients. Frequently, eye damage can worsen and turn into chronic conjunctivitis or corneal ulcers [53]. Inflammatory ophthalmological conditions include episcleritis, scleritis, and peripheral ulcerative keratitis and can greatly exacerbate ocular prognosis as they aggravate RA conditions and increase the risk of developing systemic vasculitis [54]. For this reason, at the time of the diagnosis of RA, there should be a close collaboration between the rheumatologist and the ophthalmologist. Scleritis, for example, when associated with RA, can lead to severe ocular complications and is thought to be caused by the deposition of the immune complex found in the necrotizing collagen associated with the sclera [55]. Scleritis in 40% of cases is bilateral and the necrotizing form is associated with an increase in the severity of RA; the characteristic symptom is represented by eye pain that greatly increases with eye movements [56]. Most cases of scleritis are treated with immunosuppressive drugs and have a good resolution, although some refractory cases are more aggressive and resistant to steroid therapies [57].

### 2.3. Cognitive Impairment in Rheumatoid Arthritis

Although neuropsychological damage is not usually associated with RA, recent studies have suggested the possibility of developing cognitive impairment, responsible for the alteration of daily life in patients with RA [58]. Cognitive function includes many neuropsychological domains, dealing with orientation, attention, concentration, judgment, problem-solving, memory, and visual-spatial conception [59]. More than 40% of patients with RA can generate neurological diseases, such as impairments on cognitive function, memory, verbal function, and attention alterations, and psychiatric symptoms such as anxiety and depression [60]. Current data have suggested that this cognitive decline may be due to prolonged inflammation in the brain or the high risk of cardiovascular comorbility responsible for, over time, the development of metabolic syndrome and increased inflammatory protein [61]. The cognitive impairment could also be associated with clinical characteristics (persistent pain, chronic fatigue, and sleep disorders) or with psychological comorbibities such as anxiety and depression [62]. The maintenance of cognitive function in patients with chronic diseases, such as RA, is extremely important to ensure successful performance in day-to-day activities and the management of pharmacological treatment schemes. An important clinical study was conducted in order to find the possible predictors of cognitive deterioration in patients with RA [63]. A number of physical, psychosocial, and biological parameters were included in this study. A third of patients with RA were cognitively compromised. It has also been shown that reduced education, lower income, concomitant use of glucocorticoids, and cardiovascular involvement were closely associated with high probability of cognitive impairment in people with RA.

## 3. The Pathophysiology of Endothelial Dysfunction in RA

### 3.1. RA-Associted Inflammation and Endothelial Dysfunction 

During inflammatory processes, endothelial cells activate and transform their phenotype. Activation of endothelial cells can be of two types. Type I activation is rapid and includes a transient response in which they begin to interact with leukocytes and platelets and partially lose their junctions; type II activation is slower but the effect is more persistent: it involves expression of a variety of proinflammatory cytokines, including tumor necrosis factor-α (TNF-α) and interleukin-1 (IL-1) derived from activated leukocytes [64]. Following activation, endothelial cells induce greater vascular permeability for plasma proteins, express cytokines and proinflammatory enzymes, and increase the regulation of adhesion molecules responsible for leukocyte-endothelium interactions [65]. Earlier studies have suggested that endothelial dysfunction is also characterized by the accumulation of reactive oxygen species (ROS) and variations of important modulatory compounds such as vasodilator nitric oxide (NO) [66]. Since the pathophysiological basis of RA is the inflammatory process, after assessing the main characteristics of endothelial inflammation, we want to focus attention on the involvement of inflammation of the endothelium in RA. 

Inflammatory cytokines play a key role in RA. For example, it has been recognized that TNF-α, synthesized by endothelial cells, plays a fundamental role in the destruction of the joints; furthermore, it increases cell infiltration into the synovium by enhancing chemokines expression, activating endothelial cells, and increasing angiogenesis [67]. Patients with RA are characterized, in fact, by synovium that appears pink, tending to red, due to the increase of number of blood vessels. The neo-formation of blood vessels guarantees the transport of nutrients and oxygen but also of inflammatory cells to the synovial sites and maintains, consequently, a chronic inflammatory state in RA [68]. In addition, TNF-α, in association with IL-1, causes bone damage, a distinctive feature of RA [69]. Finally, IL-1 and IL-6 act in RA patients by performing joint destruction and facilitating disease progression. In particular, a crucial role is played by IL-6 and some studies have shown that the absence of this interleukin induced complete protection against arthritis in mice. In addition, an anti-mouse monoclonal antibody IL-6 inhibited the development of arthritis in the same experimental mouse model [70]. Another action of IL-6 is that it upregulates chemokines attracting T cells and increasing cellular infiltration [71]. 

### 3.2. Endoplasmic Reticulum Stress in Endothelial Cells of Patients with RA

A correlation between endothelial dysfunction and cellular endoplasmic reticulum stress was demonstrated, for the first time, by Gargalovic et al. [72]. Furthermore, a recent study found that there is a correlation between RA and dysfunction of endoplasmic reticulum, although the mechanisms were not fully clarified [73].

In eukaryotic cells, the endoplasmic reticulum (ER) is a large, dynamic organelle that performs important functions including protein synthesis, calcium storage and lipid metabolism. A particularly important function of ER is the post-translational folding of secretory proteins in the organelle lumen. The capacity for folding proteins varies greatly among cells types and, in highly secretory and metabolic tissues, millions of proteins are processed in the ER per minute [74]. It has been estimated that about 30% of nascent proteins fail to achieve their proper conformation and, to solve this problem, misfolded/unfolded proteins activate a signal transduction pathway called the unfolded protein response (UPR). UPR activates to mitigate global protein translation, reducing the load of folding, and to improve protein folding. The UPR transducers are three transmembrane proteins: inositol-requiring enzyme-1α (IRE1α), pancreatic endoplasmic reticulum kinase (PERK), and activating transcription factor-6 (ATF6). Glucose-regulated protein-78 (GRP-78) is one of the main and most abundant ER chaperones, which binds to newly synthesized polypeptides to promote their folding and also binds to poorly folded proteins to facilitate proper folding and prevent aggregation. In addition, GRP-78 is a UPR signaling modulator, which in stress-free conditions suppresses UPR activity by binding to the three UPR transducers [75]. If ER stress cannot be resolved, the cell can go to death. A scientific study has shown that the chemical induction of reticulum stress into human aortic endothelial cells by treatment with tunicamycin, which blocks N-glycosylation reactions of proteins and reduces the folding capacity of ER, increased the expression of numerous inflammatory markers. In addition, this condition was abolished by gene silencing of UPR mediators [74]. A further in vivo study on vascular endothelium obtained from cellular biopsies showed that obese individuals with endothelial dysfunction have all the UPR sensors (PERK, IRE1, and ATF6) activated [76]. Since no study has yet examined whether chronic administration of ER stress inhibitors reduces ER stress in the vascular system, further studies would be needed.

To date, much scientific evidence has shown that there is a cross-talk between ER stress and chronic autoimmune inflammation [77,78,79,80]. It has recently been highlighted that patients with RA have high levels of anti-GRP-78 antibodies in both synovial tissue and joints contributing to the development of self-reactive T cells and increasing the production of pro-inflammatory cytokines in synovial cells. In addition, GRP-78 binds to ACPA stimulating the production of pro-inflammatory cytokines and amplifying the inflammatory cascade. Finally, GRP-78 directly upregulates migration/chemotaxis and proliferation of endothelial cells, that facilitate synovial angiogenesis [81]. Since endothelial cells have a dysregulation of the endoplasmic reticulum in inflammatory processes [82], this scenario may also occur in chronic autoimmune diseases.

### 3.3. The Role of Dysfunctional Endothelium in the Release of Neurotrophic Factors in RA

Another important factor that relates endothelial dysfunction to patients with RA is brain-derived neurotrophic factor (BDNF). BDNF belongs to the neurotrophin family which includes nerve growth factor (NGF), neurorophin-3 (NT3), and neutorophin-4 (NT4). BDNF is widely expressed in the central nervous system exercising very important functions in neuroplasticity, neurogenesis, and angiogenesis [83]. BDNF is synthesized in neurons and glia and is released to terminal axons [84]; however, its presence has also been highlighted in dendritic endings [85].

To date, it is known that BDNF is also synthesized from the endothelium. In fact, the removal of endothelial cells of cerebral capillaries from the brain has shown a clear reduction in BDNF levels in brain tissue [86]. In the scientific literature, there are innumerable data that report that RA patients have a high risk of developing cardiovascular disease (CVD) and that this predisposition is the cause of premature mortality and/or a reduced life expectancy in 40–50% of these patients [87,88]. RA-associated increased cardiovascular risk has been related to endothelial dysfunction [89]. Endothelial balance is maintained thanks to its property of releasing soluble mediators, such as nitric oxide (NO), the main regulator of vascular homeostasis, which is released in response to physiological stimuli and is the main component for proper maintenance of the endothelium [90,91].

In RA, endothelial dysfunction is responsible for the alteration of small vessels of the microcirculation, essential for supplying oxygen and nutrients to the surrounding tissues, as well as for the exchange of fluids and repair processes [92]. As has already been said, RA has also been associated with impaired cognition, alteration in logical memory, working memory, executive function, and presence of depressive symptoms. A study conducted in rats, in which RA was induced, showed an alteration of the pathway that includes BDNF and its receptor in both endothelial cells and neurons. In addition, a connection between the endothelial expression of BDNF and the ability of the cerebral endothelium to produce NO was highlighted [93]. These results suggested that impaired BDNF-dependent cognition might have an endothelial component, although the neuronal component is also crucial. 

Endothelial involvement in RA was also demonstrated by the study based on the count of the number of altered endothelial cells, which under the microscope appear visibly morphologically altered. Furthermore, the vascular endothelial adhesion molecule type 1 (sVCAM-1) and the endothelins were found to be modulated [94]. A clinical evaluation of endothelial dysfunction in RA patients was performed in a prospective study involving 44 patients who had been suffering from RA for more than 12 months [95]. In order to estimate the endothelial function, the brachial artery method was adopted, measuring the percentage change in diameter, mediated by blood flow, and the results obtained were compared to those of healthy subjects. Only 6 out of 44 patients showed normal endothelial function. This study confirmed, in clinical practice, the endothelial function as an early predictor of atherosclerosis in patients with RA. Another clinical study conducted on 68 patients with RA showed that a sedentary lifestyle worsens microvascular endothelial dysfunction [96]. Early endothelial dysfunction in RA involves impaired angiogenesis that leads to cardiovascular comorbidity.

### 3.4. Disruption of Brain Nerve Barrier (BNB) and its Role on RA-Associated Neurological Manifestation

Some studies have shown a possible relationship between neuronal endothelium and neurological manifestations in RA patients [97,98]. At the level of the nervous system, there are two important barriers. The first, the blood-brain barrier (BBB), separates the central nervous system from the systemic circulation [99]; the second, at the level of the peripheral nervous system, separates the blood from the peripheral nerves and constitutes the blood-nerve barrier (BNB) [50]. The BBB and BNB have been extensively studied in the case of neurodegenerative diseases or peripheral phenomena of neurodegeneration [100,101]. 

Endothelial dysfunction at the blood-nerve interface is involved in the onset of many disease states through three mechanisms which include:

- a reduction of NO;
- a greater expression of pro-inflammatory factors; and
- a modification of the permeability of the endothelium [100].

The first mechanism is associated with reduced production of NO which results, in pro-inflammatory situations, from an increase in the accumulation of reactive oxygen species (ROS) and from the induction of endoplasmic reticulum stress in endothelial cells [102,103,104,105]. Under physiological conditions in each cell, there is a balance between the endogenous production of free radicals and their neutralization by antioxidant systems [106,107]. However, when the accumulation of ROS becomes massive, there is a drastic reduction in the endogenous antioxidant capacity and these reactive species react with organic and inorganic molecules thus producing other radicals with a series of chain reactions. The modification of the redox state results in a reduced endothelial nitric oxide synthase (eNOS), activity, with consequent inhibition of NO formation. Among the ROS, the most dangerous species is the superoxide anion (O2^−^) which can react with O_2_ to generate peroxynitrite (ONOO^−^). ONOO^−^ is a powerful oxidizing molecule capable of altering the structure of biological macromolecules, including those involved in the synthesis of NO [108,109,110]. At the same time, the involvement of the endoplasmic reticulum leads to an increased expression of endothelin-1 and a reduction in the expression of one of the enzymes responsible for the synthesis of NO, the endothelial nitric oxide improved eNOS activity and greater vascular relaxation was observed. If endoplasmic reticulum stress is not resolvable and shows too high ROS levels and altered homeostasis of the calcium ion, the endothelial cells activate pro-apoptotic signals (JNK, p38, and caspase-9) which lead to cell death [111].

The second mechanism is activated as a direct consequence of the first. In fact, the endothelial alterations previously mentioned are accompanied by the increase of various pro-inflammatory factors. It has been shown that there is an increase in C reactive protein, IL-1β, IL-6, and tumor necrosis factor (TNF-α). Finally, an increase in pro-inflammatory molecules is associated with an increase in the permeability of the endothelium, with consequent leukocyte adhesion and migration of monocytes. Up-regulation of expression of cell adhesion molecules (CAM) occurs, such as intercellular adhesion molecule 1 (ICAM-1), vascular cell adhesion molecule (VCAM-1), and selectin E [112]. These molecules are expressed on the plasma membrane of endothelial cells and increase the affinity of the leukocytes and the weakening of the barrier, thus causing diapedesis of leukocytes in the peripheral tissues. Ultimately, the breakdown of the integrity of the BNB, as a consequence of the endothelial alteration, occurs as a result of reduced expression of the proteins of the tight junctions and the adherent junctions [100].

During the inflammatory process, a significant role is also played by estrogens and this consideration is very important due to the female population’s prevalence in developing RA. As previously reported, estrogen acts as a humoral immunity enhancer, but it is equally important to emphasize that, at the nervous level, estrogens provide a protective response [113,114]. It was highlighted, in particular, that treatment with estrogen attenuates the recruitment of adhesion molecules, monocytes, and leukocytes in the endothelium of patients suffering from neurological diseases. This evidence could explain the protective effect of estrogens in the neurological field, even if it would be advisable to carry out further and more updated studies [115].

In general, 17-βestradiol and prolactin act as a humoral immunity enhancer, while testosterone and progesterone act as natural immunosuppressants. These data can explain the different clinical patterns of RA at different stages of women’s sexual life:

- higher incidence of RA in women, during the fertile sexual period, compared to men (ratio 3:1);
- menopausal remissions in women with RA [116];
- improvements in the course of the disease in approximately 75% of women during pregnancy. It is important to underline the shift that 17-βestradiol undergoes during pregnancy. In this circumstance, the function of the sex hormone is mainly anti-inflammatory with marked inhibition of pro-inflammatory cytokines, such as tumor necrosis factor, IL-1β, IL-6, and natural killer cells [117].

## 4. The Impact of Lifestyle Changes in RA 

Sixty percent of premature deaths could be attributed to unhealthy lifestyle that includes risk factors such as poor nutrition, cigarette smoking, alcohol consumption, obesity, and physical inactivity. Indeed, a healthy lifestyle has been associated with an estimated increase in life expectancy of 7.4–17.9 years in Japan, the UK, Canada, Denmark, Norway, and Germany [118,119]. Furthermore, it has been widely acknowledged that unhealthy lifestyles are the main risk factors for various chronic diseases and premature death [120]. In patients with RA, these risk factors are associated with increase of morbidity and mortality [121]. Smoking has one of the strongest associations with poor health and it has many harmful pathological effects. However, its effects seem to depend on the quantity and time of intake; for this reason, the current epidemiological evidence is difficult to interpret. Smoking of cigarettes and other tobacco products has become a risk factor for many diseases, particularly respiratory and cardiovascular. In addition, numerous studies have established an obvious relationship between smoking and the onset of RA and stressed that smoking is the strongest known risk factor to develop RA [122]. It is also interesting to note that smokers have positive results for anti-RF and anti-ACPA autoantibodies both associated with SPRA patients. Moreover, successive studies have evidenced that the rate of SPRA diminished as a result of the reduction or suspension of the smoke. SPRA and smokers have also shown a higher risk of X-ray progression and erosive disease than non-smokers. For these reasons, it would be desirable to advise patients with RA or even suspected RA to stop or reduce their exposure to smoking [123]. 

Physical exercise consists of a planned, structured, and repetitive set of movements with the aim of improving or maintaining physical fitness. The American College of Sports Medicine states that exercise is indisputably advantageous and, in healthy condition, should be an integral part of daily life, including cardiorespiratory training, endurance, flexibility, and neuromotor exercise [124]. The European League Against Rheumatism (EULAR) recommends similar behavior for patients with RA. Some clinical studies have highlighted the benefits of exercise interventions on aerobic capacity and an improvement in muscle strength in these patients [125]. 

Alcohol is a psychoactive substance that produces addiction and causes social and economic hardships, especially in heavy drinkers. It would be recommended that patients with RA do not consume alcohol, since the main pharmacological treatments include hepatotoxic substances and alcohol could interact and increase hepatotoxicity [126]. Another important reason justifying a recommended reduced alcohol consumption in RA is the indirect effects on bone tissue, which are related to the dose and duration of consumption. While moderate consumption (1–2 drinks per day) does not appear to be harmful to bone tissues, increased consumption could irreversibly damage bones and tendons [127].

### Rheumatoid Arthritis and Nutrition: Involvement of Endothelium

In recent decades, a correlation between eating habits and the onset of inflammatory or autoimmune diseases has been increasingly highlighted [128]. This association was confirmed following the discovery that certain foods promote pro-inflammatory reactions, while other foods have been shown to have anti-inflammatory properties. Therefore, the RA trend can easily be correlated to dietary choices. A very interesting clinical study was conducted on 300 subjects suffering from RA, whose main information (personal data, drugs taken, any comorbidities present, and disease activity) were entered in a six-month register. The patients were questioned about the main effects caused by the intake of an established group of foods and whether, following the intake, the symptoms of RA had improved or worsened. The 20 foods considered belonged to two categories: (a) “potentially inflammatory” such as milk, cheese, red meat, tomato, eggplant, white potatoes, hot peppers, diet soft drinks, and beer; and (b) “potentially anti-inflammatory” such as fish, spinach, blueberries, strawberries, and chocolate. The results obtained showed that in subjects suffering from RA, the intake of blueberries and fish led to a reduction in RA symptoms, while the intake of cheese, red meat, sugar, and desserts fueled the inflammation [129]. All the clinical studies conducted in this direction have been found to agree that a Mediterranean diet can slow inflammatory-based diseases such as RA [130,131,132] The Mediterranean diet includes the consumption of high amounts of fruit, vegetables, unrefined cereals, legumes, nuts, a moderate intake of white meats, fish, dairy products, yogurt, and a reduced consumption of red meat and sugar. Olive oil it is the main source of edible fats and wine is consumed regularly, but moderately. This type of diet also provides a high ratio between monounsaturated and saturated fats [133]. It is important to emphasize that fish consumption is mainly based on those containing long-chain polyunsaturated fatty acids (omega 3) and that this extremely balanced diet is related to a reduction in the risk of RA [134]. The Mediterranean diet, therefore, has anti-inflammatory potential effect and for this reason, it is well correlated as a protective tool in all those inflammatory pathologies [135]. 

Nutrition also plays an important role in modulating endothelial function. Epidemiological studies have linked the intake of unsaturated fatty acids, such as alpha-linolenic acid and long-chain n-3 fatty acids, to lower plasma concentrations of inflammatory cytokines and endothelial adhesion molecules [136]. These alterations, as has already been pointed out, have both been considered markers of endothelial dysfunction. Consumption of a Mediterranean diet has been associated with a beneficial effect on the endothelium [137]. It is plausible to think that the Mediterranean diet, therefore, can also be beneficial in the neurological involvement of RA, although on this topic, specific studies should be developed.

The management and treatment of RA and its pathological manifestations from an economic point of view, as well as the side effects resulting from drug therapies, have pushed the current medical science to increasingly use phytocompounds and drugs of natural origin with documented anti-arthritic effect, to reduce or eliminate adverse effects [138]. A protective role of natural compounds has also been demonstrated towards RA [139] and, among these, the class of polyphenols has proven particularly effective. Polyphenols represent the most studied phytochemical compounds in the last two decades since they have shown a high positive impact on health. In particular, they have shown a protective role in many degenerative pathologies of the nervous and cardiovascular systems, in cancer, inflammatory diseases, and pain of different etiologies [140,141,142,143]. Many foods, such as fruit, vegetables, virgin olive oil, wine, cereals, spices, and dried fruit contain high amounts of polyphenols. The common chemical characteristic of polyphenols is an aromatic ring to which hydroxyl groups and other substituents are linked, although about 8000 compounds are known which differ precisely for the different substituents linked to the aromatic ring. The main function of polyphenols is to have strong antioxidant properties [144].

As already mentioned, much evidence has shown a close correlation between RA and oxidative stress [145,146]. Polyphenolic antioxidants neutralize reactive species and enhance the activity of antioxidant enzymes [147]. Resveratrol, a non-flavonoid phenol, is a substance that is naturally produced by various plants, such as vines, blackberries, and cocoa, and is endowed with protective activity against pathogens such as bacteria or fungi. It has been shown that resveratrol produces protective effects in RA, reducing the accumulation of ROS, suppressing the inflammatory response and cell proliferation, and consequently decreasing cell apoptosis in synovial tissues [148,149,150,151]. The active polyphenols contained in extra virgin olive oil (oleuropein, tyrosol, and hydroxytyrosol) also have strong anti-inflammatory, anti-oxidant, and anti-proliferative activity [152,153,154,155]. In particular, the oleuropein of olive oil [156] and hydroxytyrosol [157] have shown important protective activities in RA, through the down-regulation of many inflammatory cytokines, including TNF-α, IL-1β, and IL-6. They also reduce the expression of molecules related to inflammation pathways, such as p38, JNK, p65, and lκB-α [158,159]. Precisely for these properties, numerous chemical strategies have been developed to improve the stability and efficacy of these polyphenolic compounds [160]. Scientific data show that other substances of natural origin have protective functions against RA. Among these, we mention cranberries and curcumin [161]. Curcumin, in particular, has been shown to reduce inflammation and synovial hyperplasia in rats with induced RA [162]. In addition to these compounds, there are many other polyphenols whose antioxidant and anti-inflammatory properties are known, but which have not been directly tested in RA. One of these is represented by the polyphenolic fraction of bergamot. Bergamot (*Citrus bergamia*) is an endemic plant that grows in Calabria (Southern Italy) that has a wide range of flavonoids and glycosides, such as neoeriocitrin, neohesperidin, naringin, rutin, and poncirin. This peculiar composition provides the unique property to the bergamot compared to the other citrus fruits. Due to its peculiarity, it is employed in different formulations such as essential oil, hydro-alcoholic extract, and fruit juice. Recent scientific results have identified massive antioxidant and anti-inflammatory effects on the part of the polyphenolic fraction of bergamot, to the point that this fruit is considered a real nutraceutical [163,164,165,166]. Following these considerations, bergamot could be a promising candidate for studies of its activity on in vivo models of RA. Furthermore, the polyphenolic fraction of bergamot could also be tested in the neurological manifestations caused by RA, since, to date, no encouraging results are known from phytocompounds and nutraceuticals. 

Most polyphenols prevent oxidative stress by regulating the expression of antioxidant enzyme genes. Consequently, the consumption of polyphenols could participate in the inhibition of endothelial dysfunction by reducing ROS and inducing endothelium-dependent vascular relaxation [167,168].

## 5. Conclusions

RA is accompanied by EAMs which, in some cases, aggravate the quality of life of RA patients. Neurological manifestations of RA represent, at any stage of the disease, the major EAM of RA, with a serious impact on the prognosis, though the pathophysiological mechanisms remain unclear. Endothelial dysfunction, which represents a consistent target of RA, seems to play a crucial role as a causative mechanism leading to disruption of the BNB which, in turn, is associated to neurodegenerative disease accompanying RA. In this context, maintenance of endothelial/neuronal integrity via lifestyle and nutritional interventions represents a serious challenge for an early identification of RA-associated EAMs and for better therapeutic approach in RA patients.
